# A cost effectiveness analysis of midwife psycho-education for fearful pregnant women – a health system perspective for the antenatal period

**DOI:** 10.1186/s12884-017-1404-7

**Published:** 2017-07-11

**Authors:** J Toohill, E Callander, J Gamble, DK Creedy, J Fenwick

**Affiliations:** 1 School of Nursing and Midwifery & Menzies Health Institute Queensland, Griffith University, University Drive, Meadowbrook, 4131 Australia; 2 Australian Institute of Tropical Health and Medicine, James Cook University, Townsville, 4812 Australia; 3 Gold Coast University Hospital, Southport, 4215 Australia; 4 Office of the Chief Nursing and Midwifery Officer, Queensland Department of Health, Brisbane, 4001 Australia

**Keywords:** Childbirth fear, W-DEQ A, Cost effectiveness, Caesarean section, Midwife psycho-education

## Abstract

**Background:**

Psycho-education can reduce childbirth fear and caesarean section numbers. This study determines the cost-effectiveness of a midwife-led psycho-education intervention for women fearful of birth.

**Method:**

One thousand four hundred ten pregnant women in south-east Queensland, Australia were screened for childbirth fear (W-DEQ A ≥ 66). Women with high scores (*n* = 339) were randomised to the BELIEF Study (Birth Emotions and Looking to Improve Expectant Fear) to receive psycho-education (*n* = 170) at 24 and 34 weeks of pregnancy or to the control group (*n* = 169). Women in both groups were surveyed 6 weeks postpartum with total cost for health service use during pregnancy calculated. Logistic regression models assessed the odds ratio of having vaginal birth or caesarean section in the study groups.

**Result:**

Of 339 women randomised, 184 (54%) women returned data at 6 weeks postpartum (Intervention Group *n* = 91; Control Group *n* = 93). Women receiving psycho-education had a higher likelihood of vaginal birth compared to controls (*n* = 60, 66% vs. *n* = 54, 58%; OR 2.34). Mean ‘treatment’ cost for women receiving psycho-education was AUS$72. Mean cost for health services excluding the cost of psycho-education, was less in the intervention group (AUS$1193 vs. AUS$1236), but not significant (*p* = 0.78). For every five women who received midwife counselling, one caesarean section was averted. The incremental healthcare cost to prevent one caesarean section using this intervention was AUS$145.

**Conclusion:**

Costs of delivering midwife psycho-education to women with childbirth fear during pregnancy are offset by improved vaginal birth rates and reduction in caesarean section numbers.

**Trial registration:**

Australian New Zealand Controlled Trials Registry ACTRN12612000526875, 17th May 2012 (retrospectively registered one week after enrolment of first participant).

## Background

Worldwide 14% of women are affected by severe levels of childbirth fear [1]. While definitions of severity may vary across cultures and studies, childbirth fear at high or severe levels has a significant effect on a woman’s ability to approach birth positively and achieve an optimal birth outcome [2–4]. In resource rich countries, childbirth fear is commonly associated with maternal depression and or anxiety before [5, 6] and following birth [7]. Fear is influenced or exacerbated by a myriad of personal, social, and health system factors including a maternity milieu characterised by increasing rates of obstetric interventions. As a consequence, women may develop an exaggerated concern that birth will result in harm to themselves or to their baby [8, 9].

The overall prevalence of childbirth fear in Australian women is around 25% and as high as 32% for women having their first baby [10–12]. There is evidence that caesarean section occurs more frequently in women who are fearful of childbirth [4, 13, 14], and that caesarean section increases health costs to women and maternity systems [15, 16]. Australia has higher rates of childbirth fear compared to other developed countries [1, 10, 11], and worldwide has one of the highest rates of caesarean section [17]. Several studies have reported reduced caesarean section numbers following psycho-education interventions in women with childbirth fear [18–21] but few have reported costs associated with interventions [13, 22, 23]. We have previously reported on a cost analysis of the intervention on women’s quality of life and health service usage to six weeks post birth, demonstrating no additional cost to health services following provision of antenatal midwife-led psycho-education [23]. This current paper reports on the cost of the intervention in preventing unnecessary caesarean section from a health system perspective looking specifically at costs incurred during the antenatal period. This approach was taken to provide information on the relative costs of delivering the intervention compared to standard care for decision makers responsible for healthcare delivery.

### Aim

To determine the cost effectiveness of midwife-led psycho-education for fearful pregnant women, relative to standard care.

## Methods

Data was drawn from the BELIEF (Birth Emotions and Looking to Improve Expectant Fear) study, a randomised controlled trial of midwife telephone psycho-education for women fearful of birth. The study protocol [24] and results of the intervention have been reported previously [25].

Women (*n* = 1410) in their second trimester of pregnancy at antenatal booking clinics across three maternity hospitals in Queensland, Australia were screened for childbirth fear using the Wijma Delivery Expectancy/Experience Questionnaire (W-DEQ A) [26]. Women with high levels of fear (W-DEQ A ≥ 66) were randomised to receive telephone psycho-education at 24 and 34 weeks of pregnancy from a midwife trained in the intervention (*n* = 170) or to the control group (*n* = 169). Characteristics of the intervention and control groups are shown in Table 1. Women in the control group received usual antenatal care. Both groups also received a birth decision aid booklet. Demographic, obstetric information, birth preference and psychosocial measures were collected at recruitment and 36 weeks; with birth method and health service use returned by participants six weeks following birth. For further detail please refer to the study protocol [24].

**Table 1:** Characteristics of total sample of women in the intervention and control groups at baseline & 36 weeks

Characteristic	Intervention Group *N* = 170	Control Group *N* = 169
Age (Mean)	28.5	28.7
Level of Education Attainment		
Did not complete year 12	37 (22%)	32 (19%)
Completed year 12	49 (29%)	48 (28%)
Diploma	50 (29%)	47 (28%)
Bachelor degree	23 (14%)	33 (20%)
Postgraduate qualifications	11 (6%)	9 (5%)
BMI (Mean)	26.2	27.0
Nulliparous	96 (56%)	95 (56%)
Last birth Caesarean	27 (36%)	26 (35%)
WDEQ-A Time1 Mean (SD), range	80.0 (SD 12.4) 66–127	76.3 (SD 10.6) 66–128
36 weeks		
WDEQ-A Time2^a^ Mean (SD), range	61.0 (SD 19.7) 12–117	66.5 (SD 18.2) 22–121

^a^ *p* = 0.0037

The time frame for this economic evaluation was the duration of pregnancy, with analysis conducted from a health system perspective. The number of caesarean sections prevented (either planned or unplanned) was chosen as the focal outcome of the cost-effectiveness analysis because this was the main clinical outcome produced by a reduction in fear of birth. Increasing caesarean section rates are a large driver of health system costs, being more resource intensive than vaginal births [27]. Women were surveyed at 6 weeks postpartum about their health service use during pregnancy. All reported health service use during the antenatal period up to the time of birth were included. Unit costs were combined with women’s reported frequency of health service use during pregnancy. The unit cost assigned to each type of health service use is outlined in Table 2.

**Table 2:** Costs associated with health service use

Health service use	Unit Cost	Source
Intervention costs	$40.17	Based upon Queensland Health Midwife wages
GP Visits	$36.30	Medicare Benefits Schedule, Item 23
Obstetrician visits	$75.50	Medicare Benefits Schedule, Item 116
Midwife visits	$40.17	Based upon Queensland Health Midwife wages
Nurse visits	$40.17	Based upon Queensland Health Midwife wages
Ultrasounds	$78.52	Medicare Benefits Schedule, average of items related to ultrasounds
Hospital Admissions (Antenatal)	$1547.04	2013–14 National Efficient Price rates produced by the Independent Hospital Pricing Authority, based on AR-DRG O66A, O66B
Emergency Department visits	$570.70	2013–14 National Efficient Price rates produced by the Independent Hospital Pricing Authority, Triage Category 3 (Obstetric/Gynecology illness)

Logistic regression models were used to assess the odds ratio of having a vaginal birth and having a caesarean section for women in the intervention and control groups. The models were adjusted for women’s age and education attainment, BMI, baseline W-DEQ A score and hospital site.

The number of women needed to be treated (NNT) to prevent one caesarean section was calculated based upon the population expected event risk (PEER) for caesarean section, which was assumed to be equivalent to the Control Event Risk (CER) of women who underwent usual care, and the adjusted-odds ratio of having a caesarean section for the intervention group compared to the control group. The formula used to calculate NNT was: NNT = (1-(PEER*(1-OR))) / ((1-PEER)*(PEER)*(1-OR)) [28, 29].

The incremental cost-effectiveness ratio (ICER) was estimated from the difference between the total health care costs (cost of intervention plus other health care use) of the intervention and control groups multiplied by the number of women that needed to be treated (NNT) to prevent one caesarean section.

## Results

One hundred and eighty- four women returned data at six weeks following birth (Intervention *n* = 91; Control *n* = 93). The characteristics of this final sample included in this study are presented in Table 3.

**Table 3:** Characteristics of final sample of women with non-missing health service use data in the intervention and control groups at baseline & 36 weeks

Characteristic	Intervention Group *N* = 91	Control Group *N* = 93
Age (Mean)	29.2	29.5
Level of Education Attainment		
Did not complete year 112	14 (18%)	9 (9%)
Complete year 12	23 (25%)	23 (25%)
Diploma	25 (27%)	30 (32%)
Bachelor degree	18 (20%)	26 (28%)
Postgraduate qualifications	8 (9%)	5 (5%)
BMI (Mean)	26.9	26.1
Nulliparous	51 (56%)	53 (57%)
Last birth Caesarean	14 (18.6%)	13 (14%)
WDEQ-A Time1 Mean (SD), range	82.2 (SD13.5) 66–127	75.3 (SD9.2) 66–108
36 weeks		
WDEQ-A Time2^a^ Mean (SD), range	61.4 (SD20.6) 12–117	66.1 (SD18.5) 22–121
Smoked throughout pregnancy	5 (5.5)	2 (2.2)

^a^ *p* = 0.0465

### Relationship between W-DEQ a score at 36 weeks and birth type

Across all women the W-DEQ A score at 36 weeks gestation was generally associated with a woman’s type of birth, shown in Table 4. After controlling for age, level of education attainment and hospital site, a lower W-DEQ A score compared to higher scores at 36 weeks gestation was shown to be significantly related to the likelihood of having a vaginal birth as opposed to caesarean section (−0.02, *p* = 0.027).

**Table 4:** Relationship between birth type and WDEQ score at 36 weeks gestation, all women

Birth type	N	Mean WDEQ score	SD
Vaginal birth, unassisted	83	60.3	19.2
Vaginal birth, assisted	31	62.0	17.9
Unplanned caesarean section	39	67.3	16.7
Planned caesarean section, before labour commenced	24	67.1	25.5
Planned caesarean section, after labour commenced	7	83.8	19.5
Broad birth type			
Vaginal birth – any	114	60.8	18.8
Caesarean section - any	70	68.6	20.3

### Birth type between intervention and control groups

A larger proportion of women from the intervention group had a vaginal birth (66%) than women in the control group (58%) (Table 5). After adjusting for age, level of education attainment, BMI, baseline W-DEQ A score and hospital site, women in the intervention group had 2.34 times the odds of having a vaginal birth than women in the control group (95% CI: 1.16–4.73, *p* = 0.014). Women in the intervention group had 0.41 times the odds of having a caesarean section compared to women in the control group (95% CI: 0.20–0.85, *p* = 0.017).

**Table 5:** Type of birth across the intervention and control groups

	Intervention (n, %)	Control (n, %)
Vaginal birth, unassisted	44 (48.4%)	39 (41.9%)
Vaginal birth, assisted	16 (17.6%)	15 (16.1%)
Unplanned caesarean section	16 (17.6%)	23 (24.7%)
Planned caesarean section, before labour commenced	11 (12.1%)	13 (14.0%)
Planned caesarean section, after labour commenced	4 (4.0%)	3 (3.2%)
Broad birth type		
Vaginal birth – any	60 (65.9%)	54 (58.1%)
Caesarean section - any	31 (34.1%)	39 (41.9%)

### Cost comparison between intervention and control groups

The cost estimates are summarized in Table 6, which presents both the intervention cost and health service use cost during pregnancy. The mean ‘treatment’ cost for women receiving the intervention was AUS$72. The mean cost for health service use, excluding the cost of the intervention, was less in the intervention group (AUS$1193) than the control group (AUS$1236), however this difference was not significant (*p* = 0.78).

**Table 6:** Health service use costs per woman. All figures in 2013 Australian dollars

	Intervention (Mean, SD)	Control (Mean, SD)	Difference
Intervention costs	72 (24)	0	72
Health service use - total	1193 (891)	1236 (1264)	−43
GP Visits	193 (112)	217 (101)	−24
Obstetrician visits	144 (163)	99 (139)	45
Midwife visits	111 (92)	109 (90)	2
Nurse visits	16 (38)	25 (67)	−9
Ultrasounds	259 (141)	226 (138)	33
Hospital Admissions	238 (561)	216 (539)	22
Emergency Department visits	232 (450)	331 (777)	−99
Total costs	1265 (895)	1236 (1264)	29

Based on an odds ratio for having a caesarean section following midwife-led psycho-education, the number needed to treat (NNT) was five women treated to prevent one caesarean section (95% CI: 3–26) (Table 7). The overall intervention cost was AUS$29 more per woman than routine care. The incremental healthcare cost to prevent one caesarean section using this intervention was AUS$145 (Table 7).

**Table 7:** Cost-effectiveness estimates (95% CI) for the midwife-led psycho-education intervention

Difference in total cost ($)	AUS $29
Control event risk/population expected event risk for caesarean section	0.42
Odds ratio of having a caesarean section in the intervention group (95% CI)	0.41 (0.20–0.85)
NNT (95% CI)	5 (3–26)
Cost per caesarean section prevented	AUS $145

## Discussion

Women with high childbirth fear were randomised to receive a brief psycho-education intervention from midwives who had completed a training program developed for this purpose [24]. We have previously reported that in fearful women, two antenatal psycho-education sessions with a midwife reduced high and severe score levels [25], and recommended that regardless of severity, all women fearful of birth should receive individualised support [2]. Additionally we identified that psycho-education provided by midwives resulted in a clinically important 8% reduction in caesarean section numbers [18]. The findings of our current analysis adds to this evidence by identifying the minimal cost incurred by the health system in offering brief psycho-education by midwives. In all, the incremental cost of preventing a caesarean section was AU$145 using this intervention. The cost saving per avoided caesarean section could be between AUS$2449 to $5750.48. Refer to Table 8 for costs to the health care system per birth mode.

**Table 8:** Cost to the healthcare system of different types of birth, based on National Efficient Price weights and Australian Refined Diagnosis-Related Group Codes, 2013

Birth Type	Cost to healthcare system
Unassisted vaginal birth	AUS $5000.81
Assisted vaginal birth	AUS $8301.86
Caesarean section	AUS $10,751.29

Our findings are similar to those of Rouhe and colleagues [20] who also conducted a randomised controlled trial testing the effect of six psycho-education group sessions with a psychologist on birth outcome in a group of Finnish primiparous women. These researchers reported improved vaginal birth rates without increasing costs compared to conventional treatment [22]. Health system differences between Finland and Australia make direct comparison difficult, as do the differences in inclusion criteria between the studies. Our results however, are reassuring and demonstrate that with minimal additional cost to health services women can receive individualised midwifery support that improves emotional health, confidence, and increases vaginal birth rates in these women.

A search of the literature revealed only one other study reporting on the cost of treating childbirth fear. Sjogren and Thomassen [13], Swedish researchers, provided individualised psychological and obstetric support to 100 women, 68% of whom originally requested caesarean section. Of these women, 57% achieved a vaginal birth. The authors argued that the achieved reduction in caesarean section rates associated with counselling fearful pregnant women was cost neutral. Similarly our work demonstrated that following antenatal midwife psycho-education, women had twice the odds of achieving a vaginal birth, and that midwife psycho-education was cost effective from a health service perspective. With this intervention, five women were required to be treated, for a total cost of AUS$145, to prevent one caesarean section. In addition this outcome raises the prospect of longer term cost benefits potentially impacting on the number of women who would otherwise have a repeat caesarean section in a subsequent pregnancy. The four reported costing studies to date, including our study, show regardless of how costs are attributed, providing psycho-education treatment to women who are fearful of birth does not increase costs to health services and is likely to provide health services with cumulative savings over time.

### Implications for practice

Consistent with earlier studies indicating fearful women are more likely to experience a caesarean section, we found fear scores at 36 weeks gestation to be predictive of the woman’s birth mode, with higher scores associated to caesarean section. A recent cohort study across six European countries reported that incremental increases to antenatal fear scores are associated with the woman’s subsequent birth mode [4]. Interestingly, in our study the highest scores recorded at 36 weeks of pregnancy were by women who spontaneously laboured prior to their planned caesarean section. This is an important finding alerting maternity providers to the level of preparation and support women may require to reassure them of a safe outcome, and explaining that planning a caesarean section alone is not a solution to their fear. Swedish experts [30] have previously advised that prescription or agreement for a caesarean section does not alleviate women’s fear. Our findings highlight the need for clear birth planning and an exploration of women’s understanding of alternate pathways that might occur leading up to the birth. This preparation allows women to anticipate and plan for possible eventualities. One aspect of the BELIEF psycho-education framework [24] was for midwives to encourage women to envisage how they wanted their birth to be and also what it might look like if other factors impacted and changed their desired course. Taking this approach assisted women to think through strategies they felt they could manage whilst also providing them with a ‘Plan B’, and in some cases a ‘Plan C’ if needed.

The positive effects of this midwife intervention on women’s reduced fear levels, higher vaginal birth rates and minimal cost to health organisations to reduce caesarean section rates makes investment in midwife psycho-education attractive to health services. Additionally, combining psycho-education for women with childbirth fear and cost effective midwifery caseload care is likely to produce even higher benefits for maternity service organisations and for women with childbirth fear [31–34]. To assist screening of women with childbirth fear the validated two item Fear of Birth scale (FOBs) could also be used, as it is time-efficient for clinicians, and aligns to the widely used and accepted W-DEQ A tool [35].

## Limitations and strengths

Costs were attributed to health service use and therefore women’s personal out of pocket expenditure were not known. Records of service use was reliant on women’s self-report; however women carry a personal pregnancy health record and were not reliant on recall alone. We cannot be certain of the generalisability of the findings as approximately 30% of Australian women receive private obstetric care. We recruited from public hospitals only. Also only women who spoke English were included leaving non-English speaking women marginalised and; women of lower education and income were more likely to drop out of the study [25]. Lastly, despite a number of engagement strategies only 54% of women returned data at 6 weeks following birth, although this rate is comparable to similar studies involving fearful women [22].

Although the costs reported from this study indicate the potential for significant cost savings as a result of reduced caesarean sections, a previous study reporting the total costs of this intervention, including the costs of birth mode, did not report a significant difference in total costs between the two arms [23]. This non-significant result may be due to a low sample size yielding insufficient power to detect a difference in cost between the two groups, with the study being powered to detect a difference in fear of birth scores.

The strength of the work, however, is that participants were enrolled from three hospitals allowing for sample diversity. We also did not place exclusions on women’s participation in respect of obstetric risk (for example women of any parity, singleton and multiple pregnancies, any type of previous birth). In addition Australian women do not have routine access to treatment for childbirth fear that is available in some other countries. We can be confident, therefore, that the differences between groups were a result of inclusion in the study and were unlikely to be attributable to an alternate childbirth fear intervention. Furthermore, despite women being randomised by a computerised system, women in the psycho-education group (intervention) had higher W-DEQ A scores than controls at baseline making the study result more encouraging.

## Conclusion

Following antenatal midwife psycho-education for women with high levels of childbirth fear, vaginal birth rates improved and caesarean section rates decreased compared with women who received standard antenatal care and a decision-aid booklet. For every five women who received midwife counselling, one caesarean section was averted. The incremental healthcare cost to prevent one caesarean section using this intervention was only AUS$145. Based on the result of this study it is recommended that maternity services invest in and support midwives to deliver psycho-education to women with childbirth fear. This will afford fearful women an opportunity to develop positive feelings of anticipation for their birth and subsequently improve their chance of achieving vaginal birth.

## Abbreviations

AUS: Australia
BELIEF: Birth Emotions and Looking to Improve Expectant Fear
CS: Caesarean section
FOBs: Fear of Birth scale
ICER: Incremental cost-effectiveness ratio
NNT: Number needed to treat
PEER: Population expected event risk
W-DEQ A: Wijma Delivery Expectancy/Experience Questionnaire – Antenatal
